# Integrated Immunopeptidomic and Proteomic Analysis of COVID-19 lung biopsies

**DOI:** 10.3389/fimmu.2023.1269335

**Published:** 2023-10-20

**Authors:** Shanye Yin, Susan Klaeger, Vipheaviny A. Chea, Isabel P. Carulli, Suzanna Rachimi, Katharine E. Black, Michael Filbin, Lida P. Hariri, Rachel S. Knipe, Robert F. Padera, Jonathan D. Stevens, William J. Lane, Steven A. Carr, Catherine J. Wu, Edy Yong Kim, Derin B. Keskin

**Affiliations:** ^1^ Department of Medical Oncology, Dana-Farber Cancer Institute, Boston, MA, United States; ^2^ Harvard Medical School, Boston, MA, United States; ^3^ Broad Institute of MIT and Harvard, Cambridge, MA, United States; ^4^ Translational Immunogenomics Laboratory, Dana-Farber Cancer Institute, Boston, MA, United States; ^5^ Division of Pulmonary and Critical Care Medicine, Massachusetts General Hospital, Boston, MA, United States; ^6^ Department of Emergency Medicine, Massachusetts General Hospital, Boston, MA, United States; ^7^ Department of Pathology, Massachusetts General Hospital, Boston, MA, United States; ^8^ Department of Pathology, Brigham and Women’s Hospital, Boston, MA, United States; ^9^ Department of Medicine, Brigham and Women’s Hospital, Boston, MA, United States; ^10^ Division of Pulmonary and Critical Care Medicine, Brigham and Women’s Hospital, Boston, MA, United States; ^11^ Section for Bioinformatics, Department of Health Technology, Technical University of Denmark, Lyngby, Denmark; ^12^ Department of Computer Science, Metropolitan College, Boston University, Boston, MA, United States

**Keywords:** COVID-19 -, immunopeptidome, proteomics, HLA-I, macrophage

## Abstract

**Introduction:**

Severe respiratory illness is the most prominent manifestation of patients infected with SARS-CoV-2, and yet the molecular mechanisms underlying severe lung disease in COVID-19 affected patients still require elucidation. Human leukocyte antigen class I (HLA-I) expression is crucial for antigen presentation and the host’s response to SARS-CoV-2.

**Methods:**

To gain insights into the immune response and molecular pathways involved in severe lung disease, we performed immunopeptidomic and proteomic analyses of lung tissues recovered at four COVID-19 autopsy and six non-COVID-19 transplants.

**Results:**

We found signals of tissue injury and regeneration in lung fibroblast and alveolar type I/II cells, resulting in the production of highly immunogenic self-antigens within the lungs of COVID-19 patients. We also identified immune activation of the M2c macrophage as the primary source of HLA-I presentation and immunogenicity in this context. Additionally, we identified 28 lung signatures that can serve as early plasma markers for predicting infection and severe COVID-19 disease. These protein signatures were predominantly expressed in macrophages and epithelial cells and were associated with complement and coagulation cascades.

**Discussion:**

Our findings emphasize the significant role of macrophage-mediated immunity in the development of severe lung disease in COVID-19 patients.

## Introduction

The emergence of the severe acute respiratory syndrome coronavirus 2 (SARS-CoV-2) in late 2019 led to the current global pandemic of coronavirus disease 2019 (COVID-19), resulting in millions of deaths worldwide (1–6). Extensive characterization of the immune response to SARS-CoV-2 infection has revealed this to be a complex and dynamic process, with a wide range of clinical manifestations ranging from asymptomatic to severe and life-threatening disease (6–9). It has been suggested that the host immune response plays a crucial role in the development of severe COVID-19 disease, which is marked by an excessive inflammatory response in the lungs, leading to the development of acute respiratory distress syndrome (ARDS) and multi-organ failure (4, 5, 10–15). However, the underlying mechanisms of immune dysregulation in severe COVID-19 lung disease remain not fully understood.

Recent studies have suggested that the release of self-antigens may contribute to the immune-mediated tissue damage observed in severe COVID-19 (13, 16, 17). In autoimmune diseases, self-antigens are known to trigger an immune response, leading to tissue damage. By analogy, a similar process may occur in COVID-19, where the release of self-antigens by infected cells could have the potential to contribute to the immune-mediated tissue damage observed in severe cases. Effective antigen presentation and immune response against viral infection rely on the expression of the major histocompatibility complex (MHC) molecules, also known as human leukocyte antigen (HLA) (18, 19). Therefore, the characterization of HLA-binding peptides in COVID-19 patients may provide further understanding of the immunological processes underlying this infection and supply insights for the rational design of novel treatments.

To this end, we applied our recently developed highly sensitive method for profiling the HLA class I (HLA-I) immunopeptidome, which entails the purification of the HLA-I complex and high-resolution mass spectrometry (MS) identification of HLA-I binding peptides (20, 21), to real-life autopsy samples of COVID-19 patient lung tissues. We combined these data with global lung and plasma proteomics measurements, as well as single-cell transcriptome data to better define the pulmonary response associated with severe COVID-19 disease.

## Results

### HLA-I immunopeptidome and proteome in lethal COVID-19 lung

**Figure 1:**
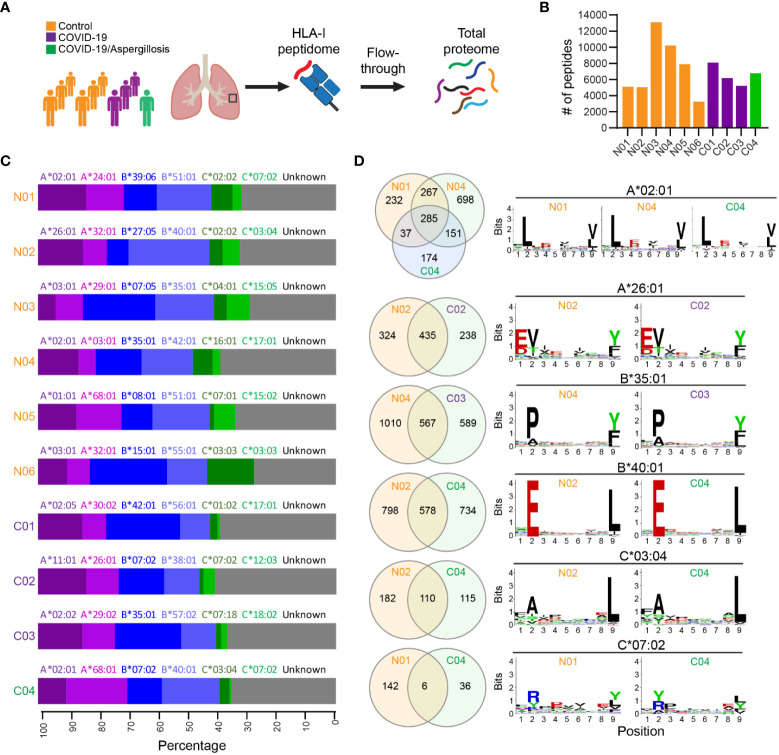
HLA-I immunopeptidome in lethal COVID-19 lung and controls. **(A)** Schematic of the experimental workflow. HLA-I proteins were immunoprecipitated (IP) from lung tissue lysates and HLA-bound peptides were identified by liquid chromatography-tandem MS (LC-MS/MS). Subsequently, the IP flowthrough was further analyzed by tandem mass tags (TMT) quantitative MS to measure global protein expression. HLA-I binding peptides and total proteins were identified and quantified in COVID-19 lung autopsy specimens (n=4) and Control lungs (n=6). Controls were lung tissues from patients undergoing transplantation for end stage lung disease. **(B)** The number of HLA-I peptides identified in each sample. **(C)** Fraction of observed peptides assigned to different HLA-I alleles using HLAthena prediction. **(D)** Venn diagram showing the common and unique peptides predicted to be associated with the same HLA allele in different samples. The Motifs of 9-mer peptide sequences were also shown.

We utilized a recently established workflow (20, 21) to investigate the HLA-I immunopeptidome and total proteome in lung autopsy specimens obtained from four fatal COVID-19 cases, one of which had COVID-19 associated pulmonary aspergillosis (**Figure 1A**). Six explanted lung tissues from patients undergoing transplantation served as non-COVID-19 controls. Demographic and clinical data for all cases were summarized in **Tables S1-3**. In total, we identified 3,245 to 13,109 HLA-I peptides across the different samples (**Figure 1B**). Notably, in contrast to cell culture infected with SARS-CoV-2 (22), we did not detect any SARS-CoV-2 peptide in COVID-19 lung samples. These findings align with previous RNA-seq investigations, which have consistently suggested minimal or absent viral transcript levels in COVID-19 lung tissue (12, 23). We benchmarked the identified peptides with known HLA binding peptides in HLA Ligand Atlas database (24), and found that 64-85% of the identified peptides had not been previously reported. We noted a consistent length distribution of HLA class I-bound peptides among the samples, with the majority of peptides being 9-mers (**Figure S1**).

HLA alleles selectively bind peptides that match a specific motif required for optimal interaction with the peptide-binding groove. To confirm that the HLA-bound peptides were indeed targets of the corresponding HLA-I alleles, we used the tool *HLAthena* (20) to predict the most likely HLA subtype associated with each peptide. In each case, 60-73% of the peptides were predicted to be targets of the corresponding HLA-I alleles, supporting the accuracy of the IP-MS method (**Figure 1C**). We found six HLA-I alleles that were shared among at least two cases (e.g., A*02:01), and investigated whether they bound the same or distinct peptides in different cases. Although many common peptides were identified, a large proportion of peptide species predicted to bind the same HLA-I allele were distinct among different samples (**Figure 1D**). This variability was unlikely to be caused solely by viral infection, as substantial differences were observed between two non-COVID-19 cases (i.e., N01 & N04) in their peptide repertoire bound to HLA-I A*02:01. We then examined the binding motifs of all 9-mer peptides for each HLA-I allele and found no significant difference in HLA binding motifs following infection, indicating that the basic mechanics of peptide presentation were not significantly altered by viral infection.

### Proteins with altered HLA-I peptide presentation in COVID-19 lung disease

**Figure 2:**
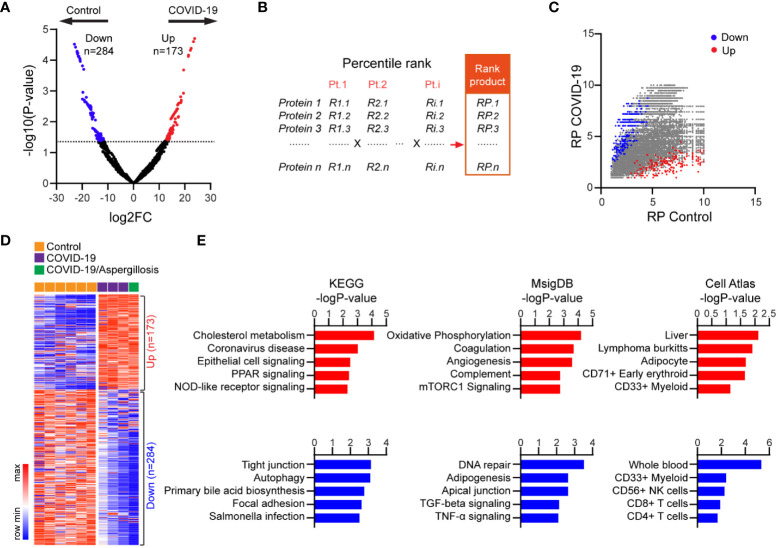
Proteins with differential HLA-I presentation in lethal COVID-19 lung versus Controls. **(A)** Volcano plot comparing HLA-I peptide presentation between lethal COVID-19 lung and Controls. The most abundant peptide was used to represent HLA presentation of the corresponding protein in each sample. MS intensity of HLA-I peptides was log-transformed and differentially presented proteins were determined by MaxQuant (p<0.05, Fold-change >2). **(B)** Schematic of the rank product-based method to evaluate HLA presentation. Proteins had been divided into 10 bins in each sample based on their rank in the abundance of the HLA peptide (e.g., top 10%, top 20%). A rank product was then generated including all samples in the group (e.g., COVID-19). **(C)** Mapping of the significantly up- or downregulated proteins identified in **(A)** into the plot of rank products. **(D)** Heatmap of HLA peptide abundance of proteins with differential HLA-I presentation in lethal COVID-19 lung versus Controls. **(E)** The enrichment of proteins with differential HLA-I presentation in KEGG pathways, MsigDB gene sets and Cell Atlas markers. The top five categories were shown, also see **Tables S5-7**.

Infection with SARS-CoV-2 can cause an overactive immune response and excessive release of cytokines (8, 13, 14, 25), leading to cell death and the release of self-antigens. We aimed to investigate if altered HLA-I peptide presentation in fatal cases of COVID-19 could be linked to key features of severe disease, such as tissue damage and cytokine storm. Using the most abundant HLA-I peptide to represent each protein in distinct samples, we identified 173 proteins with upregulated and 284 proteins with downregulated signals of HLA-I peptides in fatal COVID-19 lung versus controls (**Figure 2A**; **Table S4**).

To examine the robustness of our results, we reanalyzed our data in which we controlled for the distinct HLA alleles among different samples. Specifically, a percentile rank was assigned to individual proteins based on HLA-I peptide intensity in each case, and a rank product (RP) was generated to integrate the ranks of all samples within the same group (**Figure 2B**, see method). In this approach, proteins with low RPs were likely to have consistently high levels of HLA-I peptides in all samples. Assigning the differentially presented proteins (**Figure 2A**) to the RP plot confirmed the robustness of the changes, which were not influenced by the different HLA subtypes (**Figure 2C**). The high consistency of the HLA-I peptide changes across all samples was further demonstrated by the intensity heatmap (**Figure 2D**).

KEGG pathway analysis revealed that proteins with enhanced HLA-I peptide presentation in fatal COVID-19 lung were highly enriched for known genes involved in coronavirus disease, as expected (**Figure 2E**; **Table S5**). In addition, these proteins were most highly enriched for cholesterol metabolism, a pathway known to facilitate COVID-19 entry (26). Of note, increased antigen presentation was observed for proteins involved in epithelial signaling, PPAR signaling, and NOD-like receptor signaling, consistent with epithelial cell injury and cytokine storm (**Figure 2E**; **Table S5**). In contrast, proteins with reduced HLA-I peptides in COVID-19 were enriched for pathways such as tight junction and autophagy. In addition, the Molecular Signatures Database (MSigDB) enrichment analysis (**Figure 2E**; **Table S6**) revealed proteins with increased HLA-I peptides were enriched for oxidative phosphorylation, coagulation, angiogenesis, and complement, which are associated with intracellular oxidative stress and hyperactivation of these pathways. In contrast, proteins with reduced HLA-I peptides were enriched in DNA repair, adipogenesis, and apical junction. Notably, apical junctions are specialized structures that help to maintain the integrity and barrier function of epithelial tissues. Reduced presentation of apical junction-related peptides may suggest a disruption of these structures, leading to increased permeability and decreased barrier function. Additionally, Cell Atlas analysis revealed that proteins with increased HLA-I peptides were most enriched for liver markers, consistent with a high incidence of liver injury in severe COVID-19 cases, while proteins with reduced HLA-I peptides were enriched for CD4+ and CD8+ T cell markers, consistent with the reduced T cell counts that has been observed in patients with severe COVID-19 (23) (**Figure 2E**; **Table S7**). Overall, our findings support the notion that HLA-mediated presentation and immunogenicity plays a role in the pathogenesis of severe lung injury following COVID-19 infection.

### Changes in global protein expression in COVID-19 lung disease

**Figure 3:**
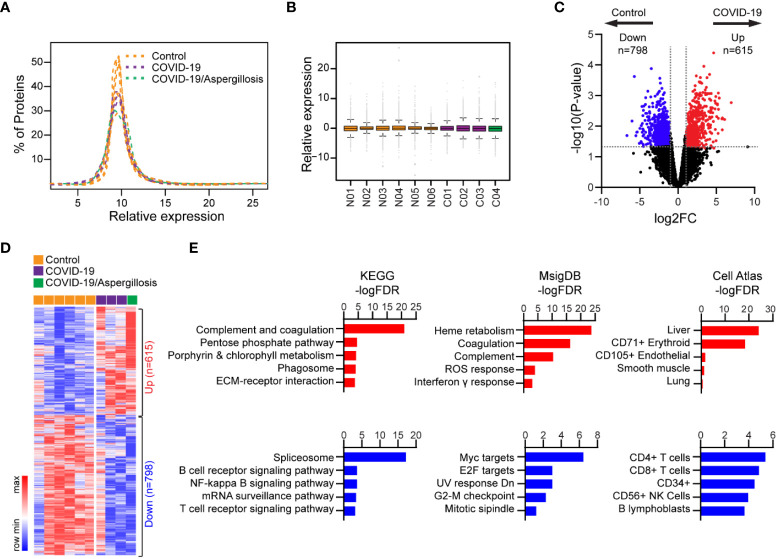
Differential protein expression in lethal COVID-19 lung versus Controls. **(A)** Histogram showing distribution of protein expression levels across different samples. **(B)** Box plot showing distribution of protein expression levels across different samples. The box shows the quartiles, the bar indicates median, and the whiskers show the distribution. **(C)** Volcano plot comparing protein expression between lethal COVID-19 lung and Controls. Foldchange>2 and P<0.05 was considered significant. **(D)** Heatmap of differentially expressed proteins in lethal COVID-19 lung versus Controls. **(E)** The enrichment of proteins with differential HLA-I presentation in KEGG pathways, MsigDB gene sets and Cell Atlas markers. The top five categories were shown, also see **Tables S9-11**.

Using Tandem Mass Tag (TMT)-based quantitative proteomics, we investigated alterations in global protein expression in lethal COVID-19 compared to controls. Our analysis quantified 8771 proteins, and their expression showed a normal distribution and similar variance across all samples following normalization (**Figures 3A, B**). We detected 798 significantly upregulated and 615 significantly downregulated proteins in COVID-19 cases versus controls (p<0.05, Fold-change>2) (**Figures 3C, D**; **Table S8**), without substantial influence of pulmonary aspergillosis on global protein expression in COVID-19 cases. By KEGG pathway analysis, the upregulated genes were most significantly enriched in complement and coagulation pathways, which is consistent with hyperactivation of immune response. In contrast, downregulated genes were significantly enriched in spliceosome and B/T cell receptor signaling pathways, indicating the commonly observed B and T cell depletion in severe COVID-19 patients (23) (**Figure 3E**; **Table S9**). Consistently, MSigDB analysis revealed that upregulated proteins were enriched in heme metabolism, coagulation, complement, ROS response and interferon γ response, while downregulated proteins were enriched in Myc/E2F targets, G2-M checkpoint, and mitotic spindle, suggesting impaired cell proliferation, which may be linked to alveolar epithelial injury and regeneration (**Figure 3E**; **Table S10**). Additionally, Cell Atlas analysis revealed that upregulated proteins were most significantly enriched for liver markers, while downregulated proteins were most significantly enriched for CD4+/CD8+ T cell markers (**Figure 3E**; **Table S11**), consistent with inadequate T cell response in the lungs of severe COVID-19 cases (27). Our quantitative proteomics data thus supports the impact of complement and coagulation pathways, B and T cell depletion, and impaired cell proliferation in severe COVID-19.

### Cellular origins of upregulated HLA-I peptides and proteins associated with COVID-19 lung disease

**Figure 4:**
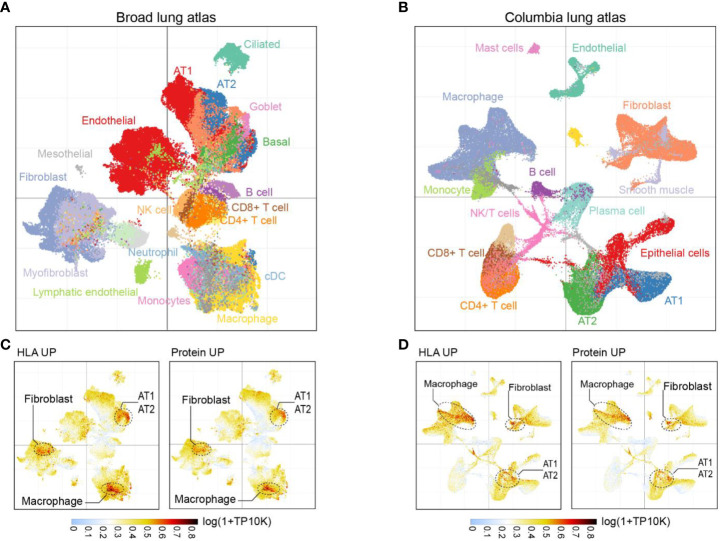
Cell subpopulations associated with HLA-I and protein signatures. **(A)** UMAP projection highlighting major parenchymal, endothelial and immune cell subsets in Broad lung atlas and **(B)** Columbia lung atlas. **(C)** Intensity map of mean expression of genes with significantly upregulated HLA-I presentation or protein expression in Broad lung atlas or **(D)** Columbia lung atlas. Gene expression was calculated by log(1+TP10K). TP10K, transcript per 10000 reads.

To investigate the cellular origins of upregulated HLA-I peptides and proteins in fatal COVID-19 patients’ lungs, we reanalyzed two external single-cell transcriptome datasets of COVID-19-affected lung tissues. The first dataset, the Broad Institute Lung Atlas (28), contains sc/snRNA-Seq data of 106,792 cells, while the second, the Columbia Lung Atlas (27), contains 116,314 snRNA-seq profiles collected from 16 and 19 COVID-19 autopsy donors, respectively. By UMAP, we annotated the major parenchymal, endothelial, and immune cell subsets (**Figures 4A, B**) and calculated the mean expression of signatures with upregulated HLA-I peptide or total protein expression (i.e., HLA UP or Protein UP) per cell. From the Broad Lung Atlas dataset, genes likely to generate more HLA-I peptides in COVID-19 were highly enriched in a subset of fibroblasts, in alveolar type I/II (AT1/AT2) cells, and in macrophages (**Figure 4C**). Similarly, genes with increased protein expression in COVID-19 were enriched in the same subset of cells, suggesting their functional relevance. Indeed, single cell analysis revealed that AT1/AT2 cells were the major cell types expressing angiotensin-converting enzyme 2 (ACE2) in the lung and thus were directly targeted by SARS-CoV-2 infection (**Figure S2A**). The elevated fibroblast signal observed in the lungs of COVID-19 patients highlights the potential connection between fibroblasts and the development of fibrosis in these individuals. Fibroblasts play a crucial role in tissue repair and wound healing, but an excessive and prolonged activation of fibroblasts can lead to the accumulation of excess collagen and the formation of scar tissue, known as fibrosis. Furthermore, our examination of multiple macrophage phenotypic markers (29, 30) revealed that the affected macrophages belonged to a unique population of CD14+CCL18+ M2c macrophages (**Figure S2B**). These results suggest that tissue injury related to fibroblast/AT1/AT2 cells and immune activation of M2c macrophages were the major sources of changes in both HLA-I peptides and protein expression in fatal COVID-19 lungs. Highly consistent results were observed in the Columbia Lung Atlas dataset (**Figure 4D**), confirming the robustness of our model.

### Lung-derived protein expression in plasma as early markers of viral infection

**Figure 5:**
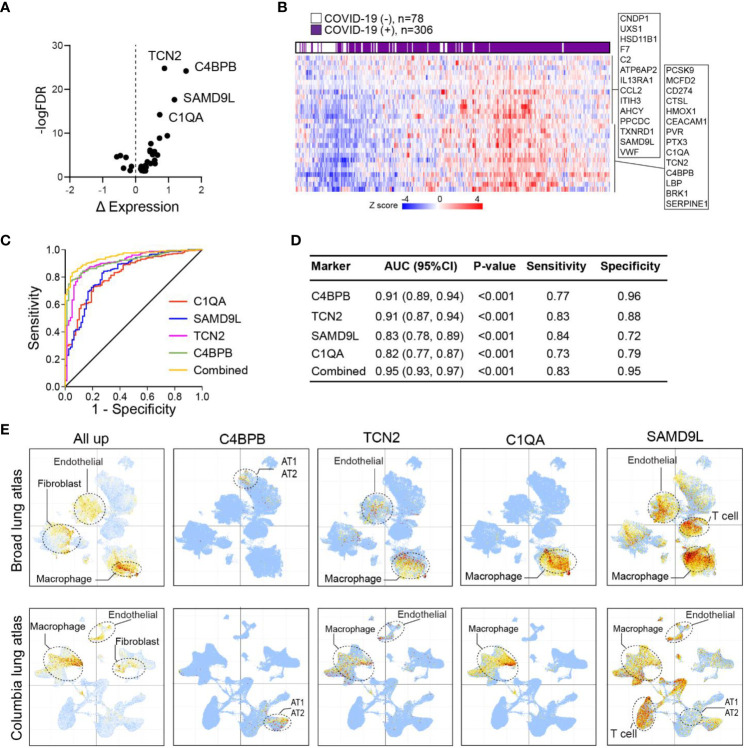
Lung proteins as early plasma markers for viral infection. **(A)** Volcano plot of differentially expressed plasma proteins based on mean normalized protein expression (NPX) values between COVID-19(+) (n=206) and COVID-19(−) patients (n=78). Only signature proteins with consistent changes in COVID-19 lung are shown. Δ NPX: COVID19(+) – COVID19(−). **(B)** Heatmap of plasma expression of upregulated protein signatures between COVID-19(+) versus COVID-19(−) patients in **(A)**. **(C)** ROC curve analysis of protein signatures predicting viral infection. Proteins with area under the ROC Curve (AUC)>0.8 are shown. **(D)** Prediction powers of different markers and their combination. **(E)** Intensity map of signature genes expression in the COVID-19 lung UMAP.

To investigate whether specific protein signatures of lung tissue injury and inflammation in COVID-19 could be detected early in circulating blood, we compared our lung tissue proteomics data with previously published plasma proteomics data collected from 309 COVID-19(+) and 78 COVID-19(-) patients on the day of hospitalization (31). The examination unveiled a set of 28 upregulated and 6 downregulated signature proteins which consistently displayed alterations in both the lung and plasma proteome of individuals with COVID-19 compared to those without the infection (**Figure 5A**). Furthermore, through unsupervised clustering analysis using plasma signals, it was evident that the 28 immune-activated proteins were effective in distinguishing infected patients from non-infected individuals (**Figure 5B**). By analysis of the receiver operating characteristic (ROC) curve for each marker, we observed that the area under the curve (AUC) for patients with severe COVID-19 ranged from 0.55 to 0.91. Notably, four markers (C4BPB, TCN2, SMD9L, and C1QA) displayed an AUC exceeding 0.8, with a combined AUC of 0.95 (**Figures 5C, D**), suggesting their potential to serve as early markers for viral infection and severe disease.

To gain insight into the cell types associated with these upregulated markers, we mapped the expression of these proteins in COVID-19 lung single-cell transcriptome data. This analysis revealed once again an overall enrichment of these markers in macrophage, endothelial, and fibroblast subsets (**Figure 5E**). Of interest, each marker displayed distinct enrichment patterns in specific cell types. Among the markers, C4BPB, which exhibited the best performance, was exclusively expressed in AT1/AT2 cells, indicating early infection and tissue damage. Additionally, TCN2 and C1QA showed predominant expression in macrophages, suggesting the involvement of macrophage activation in viral infection. In contrast, the SAMD9L gene was broadly expressed in macrophages, endothelial cells, and T cells. KEGG and MsigDB analyses highlighted the significant enrichment of these markers in the complement and coagulation cascades (**Table S12**). These findings emphasized the aberrant activation of lung-resident macrophages as a crucial feature associated with severe COVID-19.

## Discussion

While COVID-19 infected patients display a wide range of symptoms, respiratory failure remains the primary cause of mortality in severe cases. HLA-mediated immunity in COVID-19 plays a critical role in regulating immune response in the lung but is poorly understood. In this study, we present a comprehensive HLA-I immunopeptidome of fatal COVID-19 lung tissues. Integrating this data with global protein expression and single-cell data, a coherent picture emerges, such that signatures related to AT1/AT2 cell damage/regeneration and pulmonary fibrosis are hallmarks of severe COVID-19 lung disease. Additionally, immune signatures associated with M2c macrophage activation are also observed. When compared to non-COVID-19 controls, COVID-19 lungs displayed enhanced self-antigen presentation for proteins involved in cholesterol metabolism, coronavirus disease, epithelial cell signaling, oxidative phosphorylation, coagulation, angiogenesis, and complement. Our total proteome analysis also revealed hyperactivation of coagulation and complement pathways, reactive oxygen species (ROS), and interferon γ response. Thus, our results not only confirm known features of severe COVID-19 but also highlight several novel characteristics of tissue injury and the inflammatory response.

AT1 and AT2 cells, which are major components of the respiratory alveolar epithelium, express high levels of ACE2 and are the early targets of SARS-CoV-2 infection (32). Indeed, they were the only cell types with detectable ACE2 signals in single cell data of COVID-19 lung (**Figure S2A**). They form a tight barrier to prevent fluid leakage from the interstitium and vascular compartments into the alveolar space. Viral infection can induce apoptosis of these epithelial cells, causing vascular leakage, self-antigen release, and immune cell recruitment into the lungs. As a result, severe COVID-19 is characterized by endothelial injury and diffuse alveolar damage in the acute phase, followed by regeneration of injured AT1/AT2 cells and fibroblasts in the late phase, which leads to extensive pulmonary fibrosis and a loss of normal lung architecture. In line with this model, our study identified a clear signal of pulmonary endothelial cell dysfunction in severe COVID-19 lungs, along with hyperactivation of PPAR signaling, which increases the expression of death receptors and augments death receptor-induced apoptosis. We also observed downregulation of both HLA-I peptides and proteins associated with TCR signaling, suggesting functional impairment of the SARS-CoV-2-specific T cell response in severe COVID-19 patients. This lack of appropriate T cell response likely contributes to fatal outcomes (10, 23, 28).

Furthermore, comprehensive analysis of the HLA and protein signatures also revealed a significant increase in macrophages, particularly the M2c subtypes. Macrophages play a crucial role in antigen presentation and serve as the first line of defense against invading pathogens (33). Macrophages are polarized towards either proinflammatory (M1) or anti-inflammatory (M2) phenotypes in response to viral infection (29, 30, 34). Aberrantly activated macrophages have been implicated in COVID-19 complications such as acute respiratory distress syndrome, intravascular coagulation, edema, and pneumonia (35). Although the function of M2c macrophages remains largely unknown, it has been suggested that they are involved in phagocytosis of proinflammatory particles, including cellular debris and microbial products, and thus attenuate inflammation and tissue damage (36). This is consistent with the wound healing associated with AT1 and AT2 cells, supporting their roles as critical markers for severe tissue damage in critically ill patients. Interestingly, a recent study proposed that increased levels of interleukin-10 (IL-10) in COVID-19 lungs polarize alveolar macrophages into ACE2-expressing M2c-type macrophages that function as spreading vectors for viral infection, while depletion of alveolar macrophages in mouse models reduced SARS-CoV-2 pathogenicity (37). Although we did not detect clear ACE2 expression in these M2c macrophages in single-cell data, this finding provides an alternative explanation for the enrichment of M2c macrophages in severe COVID-19 and confirms the critical role of macrophages in the host response to SARS-CoV-2.

There has been increasing interest in using liquid biopsies to noninvasively assess the severity of COVID-19, and several novel diagnostic and prognostic biomarkers have been discovered (31). However, the sensitivity and specificity of these markers remain to be determined due to the complexity of the disease and small sample sizes. Our data support the hypothesis that protein markers with robust changes in the COVID-19 lung are more likely to be causally linked to the disease and can serve as early markers to predict disease severity. By cross-comparing our results with a longitudinal plasma proteomic analysis of severe COVID-19 (31), we identified 34 such lung protein signatures that could be used as early plasma markers of severe disease. For example, C4BPB is a component of the complement system, which plays a crucial role in the immune response against pathogens. It may be involved in regulating the complement response during COVID-19, potentially impacting the balance between viral clearance and inflammatory damage. C1QA is part of the C1 complex in the complement system, initiating the classical pathway. Complement activation can influence various aspects of the immune response, including inflammation and pathogen clearance. Dysregulation of the complement system has been implicated in inflammatory responses observed in severe COVID-19 cases. We acknowledge that the small number of samples in the exploratory research limits the statistical power and the results are not definitive without further replication or biological investigation. Particularly, to pinpoint markers specific to severe illness, future investigations should encompass a cohort of mild cases. Continued study of HLA immunity in conjunction with ongoing viral evolution will enhance our understanding of the disease pathogenesis and provide critical data for vaccine design.

## Materials and methods

### Patient cohort and clinical data collection

This study was a retrospective investigation conducted at Brigham and Women’s Hospital (BWH) with written consent obtained from either the patient or a legal surrogate. Inclusion criteria for COVID-19 patients consisted of SARS-COV-2 infection confirmed by PCR testing at the time of admission. Lung tissue from COVID 19 patients was obtained at the time of autopsy. The control cohort consisted of explanted lungs from patients undergoing transplantation for end stage lung disease. Systematic chart review was performed adhering to HIPA compliance. The following patient characteristics were recorded: patients: age, sex, race, ethnicity, smoking status, comorbidities, anti-viral treatments, oxygen supplementation at time of admission, length of stay, CT scan results, lung pathology, and cause of death (COVID-19 cohort) (**Tables S1–3**) . The Mass General Brigham Institutional Review Boards approved all studies involving human subjects. The control lung tissues were procured under IRB protocols 2013P002332 and 2019P003592; and Covid-19 lung tissues were procured under IRB protocol 2018P001724.

### HLA-I immunopeptidome profiling

HLA typing was performed at the Brigham and Women’s hospital tissue typing laboratory using NSG (20). Approximately 1 g of lung sample was processed in a BSL2+ laboratory under approved biosafety protocol 20-42320 of Dana-Farber Cancer Institute. Tissues were processed using 10 mL of protein lysis buffer containing 20 mM Tris (pH 8.0), 1 mM EDTA, 100 mM NaCl, 2% Triton X-100, 60 mM *n*-octylglucoside, phenylmethylsulfonyl fluoride (all from Sigma-Aldrich), and protease inhibitors (Complete protease inhibitor cocktail tablets, Roche). The tissue was disrupted using aerosol contained 50 mL Tissue grinder (Chemglass Life Sciences Ultra Tissue Grinder. Fischer Scientific). 1.5 mL lysis were transferred to Low Bind Eppendorf Microcentrifuge Tubes (Thermo Fisher Scientific). 2 uL Benzonase Nuclease were added to the tubes, and samples were processed for immune-peptidome and whole proteomics as described previously (38). One lysate sample without benzonase treatment was used for DNA extraction using QIAmp DNA Micro Kit (Qiagen).

### Whole proteome analysis

To generate whole proteome LC-MS/MS data, a 200 uL aliquot of HLA IP supernatants underwent a series of preparation steps. Initially, the sample was reduced with 5mM DTT (Pierce DTT: A39255) for 30 minutes and subsequently alkylated with 10mM IAA (Sigma IAA: A3221-10VL) for 45 minutes. Proteins were then precipitated using a methanol/chloroform-based method. After wash, the protein pellets were then resuspended in a solution of 100 mM triethylammonium bicarbonate (pH 8.5) (TEAB). Digestion was carried out using LysC (at a ratio of 1:50) for 2 hours on a shaker (1000 rpm) at 25°C, followed by overnight digestion with trypsin (also at a ratio of 1:50). The resulting samples were acidified to achieve a final formic acid concentration of 1% and then dried. To reconstitute the samples for analysis, they were dissolved in a solution containing 4.5 mM ammonium formate (pH 10) in 2% (vol/vol) acetonitrile. The peptides were labeled with TMT6 (Thermo Fisher Scientific), and the LC-MS/MS analysis was performed on a Thermo Orbitrap Exploris 480 (Thermo Fisher Scientific) using data-dependent acquisition (MS2 isolation width 0.7 m/z, top 20 scans, collision energy 30%).

### HLA-I antigen presentation prediction

Following MS identification of HLA-I binding peptides, the specific HLA subtype associated with each peptide was predicted using HLAthena, a prediction tool trained on endogenous LC-MS/MS-identified epitope data for 31 HLA-A, 40 HLA-B and 21 HLA-C alleles (20). Known HLA binding peptides were obtained from the HLA Ligand Atlas database (24).

### MS data analysis

MS intensity of HLA-I peptides were log transformed and the most abundant peptide, if multiple peptides were identified, was used to represent HLA presentation of the specific protein. Differentially presented proteins between COVID-19 lung versus Control were determined by MaxQuant (v2.4). Those with Fold-change>2 and adj. p value<0.05 were considered significant. Alternatively, to control for the differences in HLA alleles, each protein was assigned a percentile rank based on the maximal abundance of the HLA-I peptide in each same sample (i.e., R1.1, see **Figure 2B**), and the ranks of different samples within the same group (e.g., COVID-19) were integrated by rank product (e.g., R1.1×R2.1×… Ri.1=RP.1). Proteins had been divided into 10 bins in each sample based on their rank in the abundance of the HLA peptide (i.e., top 10%, top 20%). Pathway analyses were performed using Enrichr (39), with the KEGG pathway gene sets, the MsigDB hallmark gene sets and Cell Atlas marker gene sets. All plots were generated using Graphpad Prism software (v10).

### Single-cell data analysis

Processed single nucleus and single cell transcriptomic data of COVID-19 lung samples were obtained from the Broad Lung Atlas (GSE162911) and the Columbia Lung Atlas (GSE171524). After normalization, expression level of each gene within individual cells was calculated by log(1+TP10K) (TP10K, transcript per 10000 reads), and the mean expression of genes within a specific group (e.g., upregulated proteins in COVID-19 lung vs. Control) was then assigned to each cell.

### Cross comparison with plasma proteomics

Raw and processed plasma proteomics data collected from 309 COVID-19(+) and 78 COVID-19(-) patients were described previously (31). Normalized protein expression (NPX) involved Olink’s relative protein quantification unit on a log2 scale and values are calculated from the number of matched counts on the next generation sequencing run. Differentially expressed plasma proteins by COVID-19 status were determined by a linear model fitting each Olink protein, with COVID-19 status as a main effect and putative confounders as covariates, as described in the paper.

### Statistical analysis

Statistical analysis was performed using GraphPad Prism 10.

## Data availability statement

The original contributions presented in the study are included in the article/**Supplementary Material**, further inquiries can be directed to the corresponding author/s. Processed lung HLA and proteomics data are provided in thesupplementary tables of the paper, and raw data are available inMassIVE database (MSV000093012).

## Ethics statement

The studies involving humans were approved by Brigham and Women’s Hospital (BWH) ethics committee. The studies were conducted in accordance with the local legislation and institutional requirements. The participants provided their written informed consent to participate in this study.

## Author contributions

SY: Conceptualization, Data curation, Formal Analysis, Funding acquisition, Investigation, Methodology, Project administration, Resources, Software, Supervision, Validation, Visualization, Writing – original draft, Writing – review & editing. SK: Data curation, Methodology, Writing – review & editing. VC: Data curation, Investigation, Writing – review & editing. IC: Investigation, Resources, Writing – review & editing. SR: Methodology, Resources, Writing – review & editing. KB: Methodology, Resources, Validation, Writing – review & editing. MF: Conceptualization, Data curation, Funding acquisition, Investigation, Methodology, Project administration, Resources, Supervision, Writing – review & editing. LH: Writing – review & editing. RK: Resources, Writing – review & editing. RP: Data curation, Writing – review & editing. JS: Resources, Writing – review & editing. WL: Investigation, Writing – review & editing. SC: Data curation, Methodology, Supervision, Writing – review & editing. CW: Conceptualization, Data curation, Funding acquisition, Methodology, Project administration, Resources, Supervision, Writing – review & editing. EK: Conceptualization, Data curation, Project administration, Resources, Supervision, Writing – review & editing. DK: Conceptualization, Data curation, Formal Analysis, Funding acquisition, Investigation, Methodology, Project administration, Resources, Software, Supervision, Validation, Visualization, Writing – original draft, Writing – review & editing.

## Supplementary material

Supplementary Figure 1 Length distribution of HLA-I peptides in each sample.

Supplementary Figure 2 Intensity map of gene markers in the UMAPs. **(A)** Expression map of ACE2.**(B)** Expression map of different macrophage markers. We used mean expression of two well-established markers to specify each macrophage subtypes, as specified in the figure (e.g., CD86 and TNF for M1 macrophage).
