# Supplementary material for: Integrated Immunopeptidomic and Proteomic Analysis of COVID-19 lung biopsies
Source: Front Immunol. 2023 Oct 20;14:1269335. doi: 10.3389/fimmu.2023.1269335 (PMC10628763; doi:10.3389/fimmu.2023.1269335)
Supplement: Supplementary file 1 [file DataSheet_1.docx]

| **Table S1: Demographics of Non-COVID-19 controls and COVID-19 patients** | | |
| --- | --- | --- |
|  | **Controls***  **N=6** | **COVID 19 Patients**  **N=4** |
| **Age, mean, (SD), yr** | 60 (4.7) | 70 (16.6) |
| **Gender, Male, n (%)** | 3 (50) | 2 (50) |
| **Race** |  |  |
| White | 5 (71) | 3 (75) |
| Black | 1 (29) | 1 (25) |
| **Smoking hx, n (%)** | 4 (76) | 3 (75) |
| **Oxygen use, L/min** |  |  |
| Pre-admission | 6 (3.2) | 0 |
| **Prior lung disease** |  |  |
| ILD**^+^** | 5 (71) | 0 |
| COPD | 1 (29) | 0 |
|  |  |  |
| **Comorbidities** |  |  |
| Diabetes | 1 (15) | 3 (75) |
| HTN | 2 (33) |  |
| BMI, mean (SD) | n/a | 30.4 (5.4) |
| CAD |  |  |
| Immunosuppression |  | 1 (25) |
| Autoimmune disease |  | 1 (25) |

* End stage lung disease

^+^ IPF (n=2), RA-ILD (n=1), SSc (n=1), A1AT/COPD (n=1), CPFE (n=1)

**Table S2A. Clinical course of COVID-19 patients.**

| Patient ID | Time from symptom onset to death (d) | Hospital length of stay (d) | ICU length of stay (d) | Time on mechanical ventilation prior to death (d) | High resolution chest CT | Anti-viral or glucocorticoid steroid treatment | Cause of death |
| --- | --- | --- | --- | --- | --- | --- | --- |
| 1 -  v38 | 28 | 23 | 20 | 4 | Consolidation, ground glass opacities | None | Respiratory failure |
| 2 -  v39 | 50 | 49 | 26 | 2 | Consolidation, ground glass opacities, mild traction bronchiectasis | Remdesivir | Respiratory failure |
| 3 -  v43 | 20 | 5 | 4 | 0 | Consolidation, ground glass opacities. septal thickening, pleural effusions, cardiomegaly | Remdesivir, dexamethasone | Respiratory failure |
| 4 - v44 | 16 | 13 | 12 | 12 | No CT | None | Respiratory failure |

D, days; ICU, intensive care unit.

**Table S2B. Clinical blood laboratory values for COVID-19 patients.**

Red denotes values outside of the reference range.

| Patient ID | Absolute cell count  (1,000s / uL) | | | | | | Liver function tests | | |
| --- | --- | --- | --- | --- | --- | --- | --- | --- | --- |
|  | PMN | L | M | Eos | Baso | Plt | AST / ALT | AP | tBili |
| Reference range | *1.9-7.6* | *0.7-4.1* | *0.16-1.1* | *0.0-0.50* | *0.0-0.15* | *150-450* | *10-50 U/L* | *35-130 U/L* | *0.0-1.0mg/dL* |
| 1 - v38 | 17 | 0.50 | 7.0 | 0.01 | 0.02 | 213 | 37 / 50 | 212 | 0.2 |
| 2 - v39 | 15 | 0.65 | 0.86 | 0.00 | 0.00 | 135 | 49 / 69 | 132 | 1.5 |
| 3 - v43 | 17 | 0.17 | 0.00 | 0.00 | 0.00 | 66 | 50 / 18 | 82 | 1.8 |
| 4 - v44 | 12 | 0.62 | 0.28 | 0.12 | 0.02 | 213 | 25 / 24 | 97 | 1.6 |

PMN, neutrophils; L, lymphocytes; M, monocytes; Eos, eosinophils; Baso, basophils; Plt, platelets; AST, aspartate aminotransferase; ALT, alanine transaminase; AP, alkaline phosphatase; tBili, total bilirubin.

**Table S2C. Secondary infection in COVID-19 patients.**

| Patient ID | Clinical suspicion of secondary infection | Last blood culture  (Time prior to death) | Last respiratory culture  (Time prior to death in days) |
| --- | --- | --- | --- |
| 1 - v38 | Absent | Negative  (3d) | Oral flora#  (7d) |
| 2 - v39 | Present | Negative  (1d) | Oral flora#  Candida albicans#  **Aspergillus fumigatus**  **Pseudomonas aeruginosa%**  (1d) |
| 3 - v43 | Absent | n/a | Oral flora#  (1d) |
| 4 - v44 | Absent | Negative  (0d) | Candida albicans#  Coagulase negative staphylococcus#  (4d) |

#Oral flora, Candida albicans, and coagulase negative staphylococcus are considered contaminants and nonpathogenic organisms; these organisms are not evidence of a secondary infection.

%Pseudomonas aeruginosa was absent from prior respiratory cultures and so more likely represented an acute bacterial superinfection rather than chronic colonization of the respiratory tract.

**Table S3A. Lung Pathology of Non-COVID-19 control patients**

|  | Pathological diagnosis |
| --- | --- |
| **Control patient ID** |  |
| 1 | Alpha1-antitrypsin-related emphysema |
| 2 | Rheumatoid arthritis-related ILD |
| 3 | Pulmonary capillaritis,  pulmonary veno-occlusive disease, pulmonary artery hypertension |
| 4 | scleroderma/NSIP/PAH |
| 5 | Usual Interstitial Pneumonia (UIP)  [Idiopathic pulmonary fibrosis, IPF] |
| 6 | bullous emphysema, airway centered fibrosis, smoking related-ILD |

**Table S3B. Lung pathology of COVID-19 patients.**

| Patient ID | Microanatomy | Pathological diagnosis | Pathological phenotype# |
| --- | --- | --- | --- |
| 1 - v38 | Diffuse alveolar acute and organizing damage,  Patchy fibrosis with honeycombing  Chronic bronchiolitis | Acute and organizing  diffuse alveolar damage | Fibrotic without coagulopathy or hemorrhage |
| 2 - v39 | Fungal tracheobronchitis and bronchopneumonia,  Mucosal necrosis and inflammation    Fibrosis, cystic and post obstructive disease  Diffuse, necrotizing pulmonary hemorrhage without microthrombi  Hemosiderin-laden macrophages  Pulmonary arterial hypertension (PAH) | SARS-CoV-2 pneumonia and likely ventilator-induced lung injury without diffuse alveolar damage  Aspergillus pneumonia | Enhanced hemorrhage |
| 3 - v43 | Diffuse alveolar damage  Diffuse multifocal microthrombi  Mild emphysematous changes | Acute and organizing  diffuse alveolar damage | Enhanced coagulopathy |
| 4 - v44 | Organized diffuse alveolar damage  Acute bronchopneumonia with abundant neutrophils  Diffuse multifocal microthrombi  Mild emphysematous changes | Organizing  diffuse alveolar damage | Mixed (immune infiltration and enhanced coagulopathy) |

#Classification by the phenotypes introduced by Valdebenito et al.(1): phenotype 1: enhanced coagulation/hemorrhage; phenotype 2: immune infiltration with minimal hemorrhagic events; or phenotype 3: mixed conditions.

1. Valdebenito S, Bessis S, Annane D, Lorin de la Grandmaison G, Cramer-Borde E, Prideaux B, Eugenin EA, Bomsel M. COVID-19 lung pathogenesis in SARS-CoV-2 autopsy cases. *Front Immunol* (2021) 12:735922. Doi:10.3389/fimmu.2021.735922
