# Supplementary material for: Integrated Immunopeptidomic and Proteomic Analysis of COVID-19 lung biopsies
Source: Front Immunol. 2023 Oct 20;14:1269335. doi: 10.3389/fimmu.2023.1269335 (PMC10628763; doi:10.3389/fimmu.2023.1269335)

**Supplementary Figures**

**Figure S1.** Length distribution of HLA-I peptides in each sample.


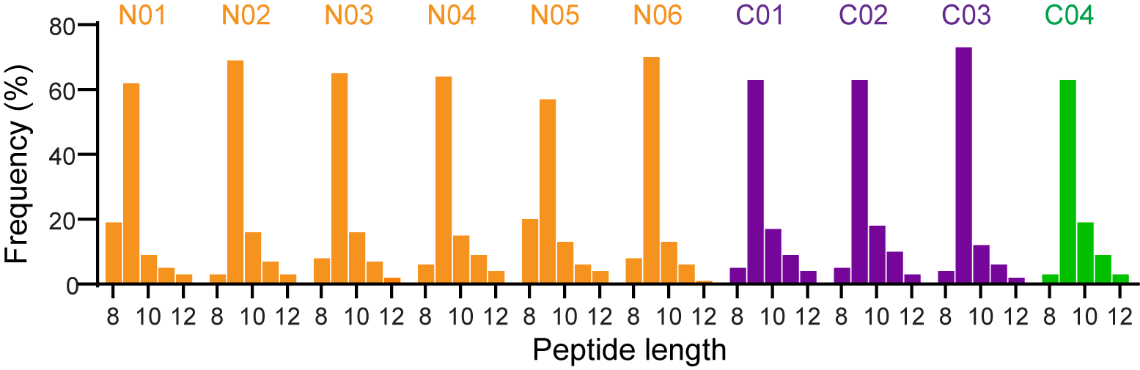


**Figure S2. Intensity map of gene markers in the UMAPs.** (A) Expression map of ACE2. (B) Expression map of different macrophage markers. We used mean expression of two well-established markers to specify each macrophage subtypes, as specified in the figure (e.g., CD86 and TNF for M1 macrophage).


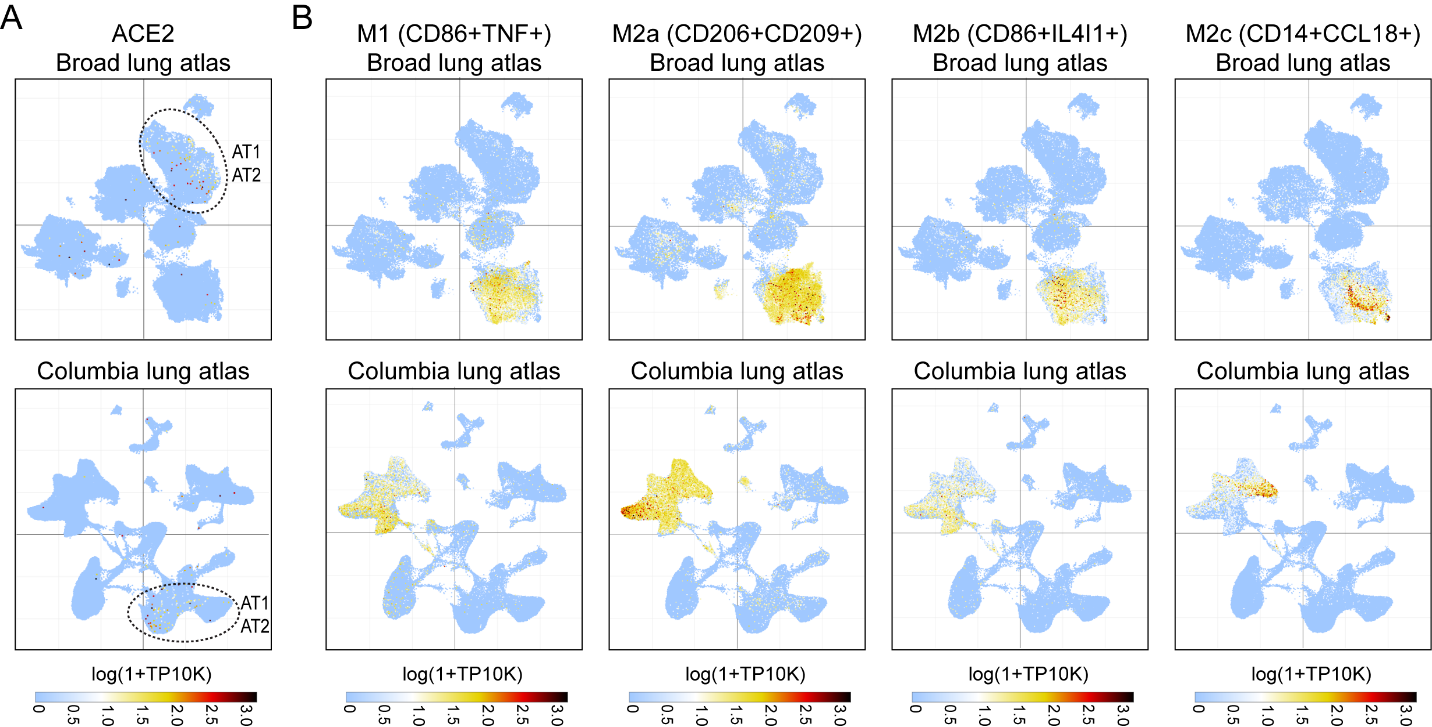

Supplement: Supplementary Figure 1 — Length distribution of HLA-I peptides in each sample. [file DataSheet_3.docx]
